# *Rax-CreER^T2^* Knock-In Mice: A Tool for Selective and Conditional Gene Deletion in Progenitor Cells and Radial Glia of the Retina and Hypothalamus

**DOI:** 10.1371/journal.pone.0090381

**Published:** 2014-04-03

**Authors:** Thomas Pak, Sooyeon Yoo, Ana M. Miranda-Angulo, Hong Wang, Seth Blackshaw

**Affiliations:** 1 Department of Neuroscience, Johns Hopkins University School of Medicine, Baltimore, Maryland, United States of America; 2 Department of Ophthalmology, Johns Hopkins University School of Medicine, Baltimore, Maryland, United States of America; 3 Department of Molecular Biology and Genetics, Johns Hopkins University School of Medicine, Baltimore, Maryland, United States of America; 4 Department of Neurology, Johns Hopkins University School of Medicine, Baltimore, Maryland, United States of America; 5 Center for High-Throughput Biology, Johns Hopkins University School of Medicine, Baltimore, Maryland, United States of America; 6 Institute for Cell Engineering, Johns Hopkins University School of Medicine, Baltimore, Maryland, United States of America; 7 Institute of Medical Research, School of Medicine, Universidad de Antioquia, Medellín, Colombia; Seattle Children's Research Institute, United States of America

## Abstract

To study gene function in neural progenitors and radial glia of the retina and hypothalamus, we developed a *Rax-CreER^T2^* mouse line in which a tamoxifen-inducible Cre recombinase is inserted into the endogenous *Rax* locus. By crossing *Rax-CreER^T2^* with the Cre-dependent Ai9 reporter line, we demonstrate that tamoxifen-induced Cre activity recapitulates the endogenous *Rax* mRNA expression pattern. During embryonic development, Cre recombinase activity in *Rax-CreER^T2^* is confined to retinal and hypothalamic progenitor cells, as well as progenitor cells of the posterior pituitary. At postnatal time points, selective Cre recombinase activity is seen in radial glial-like cell types in these organs – specifically Müller glia and tanycytes – as well as pituicytes. We anticipate that this line will prove useful for cell lineage analysis and investigation of gene function in the developing and mature retina, hypothalamus and pituitary.

## Introduction

The mammalian central nervous system (CNS) is comprised of hundreds of distinct neuronal and glial subtypes [1]–[3], and the genetic mechanisms that guide the differentiation of these cell types from neuronal progenitors are still poorly understood. In recent years, the use of mice carrying targeted conditional mutations has led to a considerable advance in our understanding of this process. The retina is a readily accessible and tractable system for studying CNS cell subtype specification. It is comprised of three cellular layers, each of which contains seven major cell types. Individual cell types can be readily identified based on morphological and molecular criteria, and each is specified from retinal progenitor cells during a discrete temporal window.

A number of mouse lines were generated in the last 15 years that use the regulatory regions of retinal progenitor-specific genes to drive expression of Cre recombinase, allowing for the selective manipulation of gene function in retinal progenitors [1]–[7]. However, each of these genetic lines has a number of inherent features that limits their usefulness for studies of gene function during retinal development. Several, such as *Chx10-Cre* and *Pax6-αCre*, are active only after neurogenesis has been initiated at approximately embryonic day (E)11, and are thus not useful for studying the genetic control of earlier stages of retinal development [4], [5]. Other lines, such as *Dkk3-Cre* and *Nes-CreER^T2^*, are active in many other CNS regions [6], [7]. Several others, such as the *Six3-Cre* and *Rax-Cre* transgenic mice show selective retinal and ventral forebrain expression starting from E8.5 but are constitutively active, making it impossible to selectively study gene function at different developmental time points [8]–[10]. The lone exception is the *Rax-IRES-tTA-tetO-Cre* line [11], which expresses Cre from the 3′ end of the endogenous *Rax* transcript in a doxycycline-repressible manner. However, these animals show ectopic Cre expression in the developing heart, as well as variable levels of microphthalmia, making them less than ideal for certain applications. Furthermore, the need to continuously supply doxycycline to maintain repression of Cre expression imposes an additional burden in maintaining this line.

We sought to build on these previous efforts to address the still unmet need for a chemically-inducible Cre line that is selectively expressed in retinal progenitors. We generated a mouse line in which tamoxifen-inducible Cre recombinase was inserted into the endogenous *Rax* locus. Since *Rax* mRNA is also expressed in hypothalamic progenitors, we expect that these animals will also be useful for studying gene function in the developing hypothalamus, a topic which has recently begun to attract considerable attention [12]–[16]. In addition, in these animals *Rax* mRNA is expressed in cells of the developing neurohypophysis or posterior pituitary [17]. In adult animals, *Rax* mRNA is selectively expressed in hypothalamic tanycytes [18], a radial glial-like cell type that have been implicated in control of energy balance and metabolism [19]–[21], and have also been found to act as adult neural progenitors [18], [22], [23]. There are few useful Cre lines available for studying hypothalamic development, and no tanycyte-specific Cre lines been reported to date [7], [22], [24]. Mice that are heterozygous for null mutations of *Rax* show no obvious defects in either retinal or hypothalamic development, and only exhibit some mild changes in tanycyte gene expression and barrier function [25].

## Results

**Figure 1:**
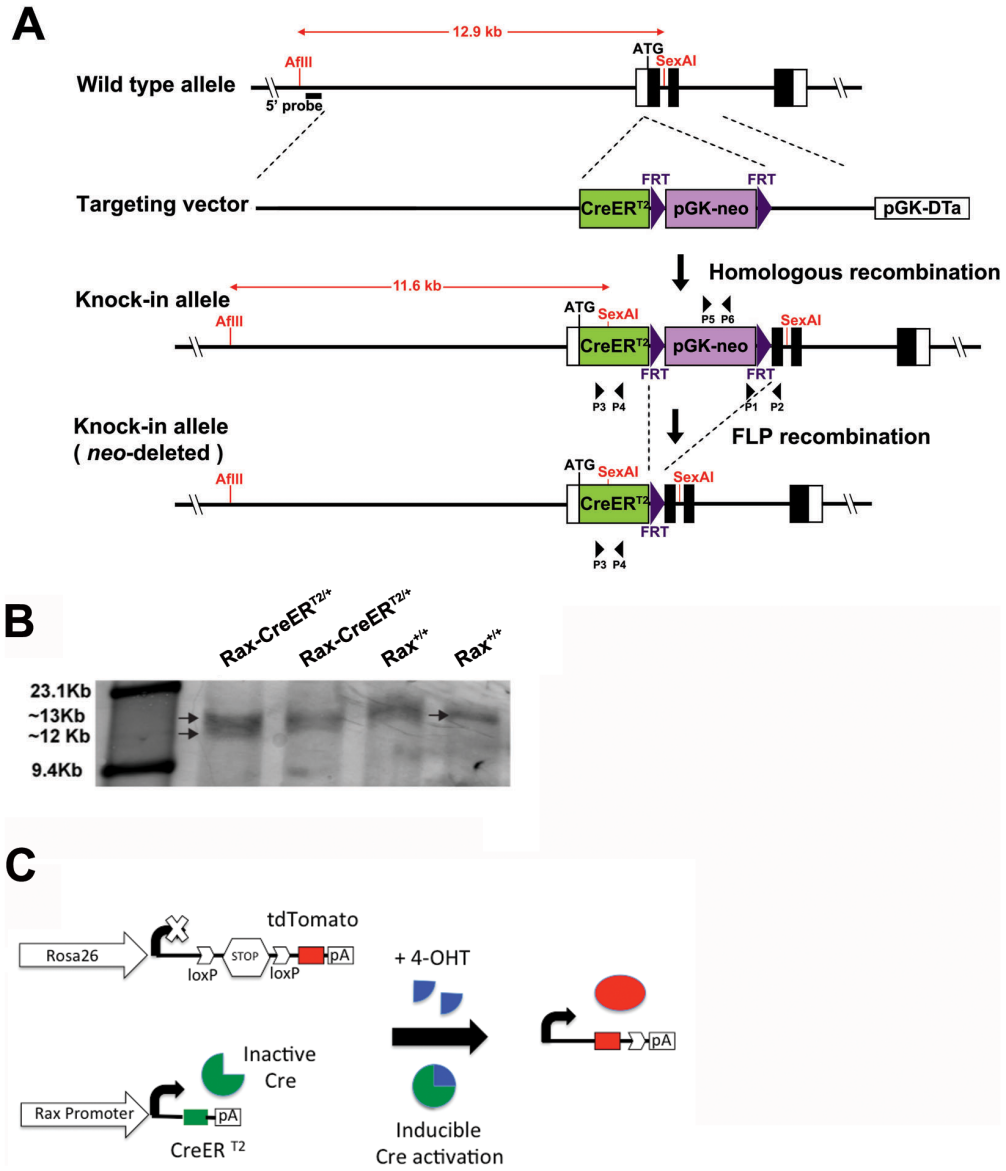
(A) Targeting strategy for generating mice in which tamoxifen-inducible Cre is expressed from the endogenous Rax locus (*Rax-CreER^T2^*). The targeting vector containing a tamoxifen-inducible Cre (CreER^T2^) and pGK-neo cassette flanked with Frt sites was inserted six nucleotides downstream from the start of the initiator codon of the endogenous *Rax* locus. After homologous recombination, the correct 3′ integration was tested in embryonic stem cells (ES) by PCR using a forward primer (P2) inside the second intron of *Rax* gene and a reverse primer (P1) in the neo cassette generating a 3 kb fragment. The Rax-CreER^T2^-neo mice were later mated with FLPeR mice to remove the neo cassette. Frt, Flp recombinase targets. (B) Correct insertion was confirmed by Southern blot using a 5′ probe which hybridized upstream of the long arm of double digested (AfIII and SexAI) DNA from ES cells. Duplicate samples of heterozygous and wildtype mice are shown. (C) Schema for *Rax*-regulated Cre-mediated Tdtomato expression induced by 4-OHT.

To generate *Rax-CreER^T2^* knock-in mice, we constructed a targeting vector in which a CreER^T2^cassette was inserted immediately downstream of the initiation methionine of the endogenous *Rax* gene, followed by a pGK-neo sequence flanked by Frt sites for selection on targeted ES cells (Figure 1A). Southern blot analysis performed using a 5′ probe external to the 7.5 kb long left arm of the targeting construct showed the presence of both 11.6 kb and 12.9 kb bands in AflII/SexAI-digested genomic DNA, while wildtype ES cells exhibited only an 11.6 kb band (Figure 1B). Following mating to FLPeR mice [26], removal of the pGK-neo cassette was confirmed using PCR analysis (data not shown).

*Rax-CreER^T2^* mice were then mated to *R26-CAG-lox-stop-lox-TdTom* (*Ai9*) mice, which express the red fluorescent Tdtomato protein under the control of the ubiquitous CAG promoter, following Cre-dependent excision of a transcriptional stop cassette (Figure 1C) [27]. We first determined whether adult animals that had not been treated with 4-hydroxytamoxifen (4-OHT) during any stage of development exhibited any evidence of Cre activity. Aside from a few rare Tdtomato-positive cells in the hippocampus and hypothalamus, no evidence for Cre activity was observed in the absence of 4-OHT (data not shown).

**Figure 2:**
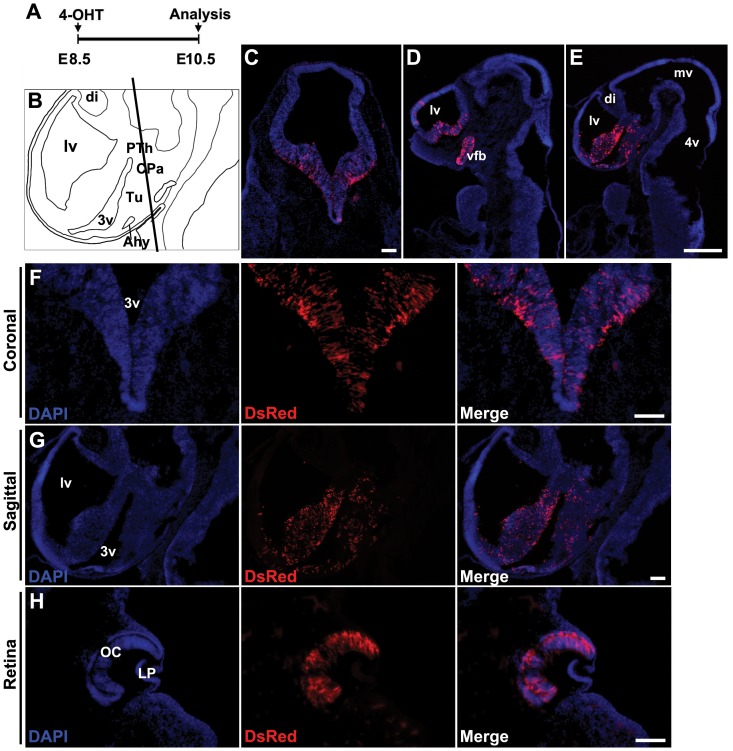
Tdtomato reporter expression is seen in neural progenitors of the ventral forebrain and retina in *Rax-CreER^T2^;Ai9* mice following 4-OHT induction at E8.5. (A) Diagram of experimental schematic. E8.5 timed pregnant mice received 1.5 mg of 4-OHT through oral gavage and were sacrificed at E10.5. (B) Anatomical structure of sagittal section. lv, lateral ventricle; di, diencephalon; 3v, third ventricle; PTh, prethalamus; CPa, caudal paraventricular nucleus area; Tu, tuberal region; Ahy, anterior hypothalamus. (C) Coronal sections corresponding to the line in (B). (D and E) Sagittal sections of *Rax-CreER^T2^;Ai9* mice. vfb, ventral forebrain; mv, mesencephalic vesicle; 4v, fourth ventricle. (F and G) High power images of (C) and (E) respectively. (H) Coronal view of retina. OC, optic cup; LP, lens pit. Tdtomato signal was boosted with anti-DsRed immunolabeling. Red fluorescence exhibited mosaic pattern limited to ventral regions of the telencephalon and diencephalon (C–G), and as robust expression was detected in neuroretina (H). Sections were counterstained with DAPI (blue). Scale bars: 100 µm (C, F–H), 200 µm (D, E).

To determine whether Cre activity could be selectively induced in *Rax*-expressing progenitors during early stages of CNS development, we delivered a single oral gavage treatment of 4-OHT delivered at E8.5, and analyzed Tdtomato expression at E10.5 (Figure 2A). We observed intense mosaic expression of Tdtomato in neural progenitors of the ventral forebrain, and labeled cells were detected in both the telencephalon and diencephalon (Figure 2C–G). Retinal progenitors are also robustly labeled (Figure 2H). No other cell types were labeled. This pattern of Cre activity corresponds precisely to the previously reported domains of *Rax* mRNA expression at E8.5 [17], [28], and confirms the specificity of Cre activity. Table 1 lists the dose of 4-OHT administered and the number of *Rax-CreER^T2^;Ai9* mice examined for this and all other experiments in this study.

**Table 1:** Summary of 4-OHT experiments described in the study.

*Time points for 4-OHT induction→analysis*	*Dose of 4-OHT*	*Delivery method*	*Number of RaxCreER^T2^:Ai9 animals tested*	*Corresponding Figure*
**E8.5→E10.5**	1.5 mg	Gavage into mother	3	Figure 2
**E10.5→E12.5**	1.5 mg	Gavage into mother	2	Figure 3
**E12.5→E14.5**	1.5 mg	Gavage into mother	3	Figure 4
**E16.5→E18.5**	1.5 mg	Gavage into mother	4	Figure 5
**P7→P9**	1.0 mg	Intraperitoneal injection	2	Figures 6 and 8
**P28→P31**	1.5 mg	Intraperitoneal injection	3	Figures 6, 8, and 9
**P50→P53**	2.0 mg	Intraperitoneal injection	3	Figures 6 and 8
**P18,19,20,21,22→P23**	0.3 mg, 0.3 mg, 0.3 mg, 1.0 mg, 1.0 mg	Intraperitoneal injection	1	Figure 7
**P5,7,9→P15**	0.3 mg, 0.3 mg, 0.3 mg	Intraperitoneal injection	2	Figures 7 and 9

**Figure 3:**
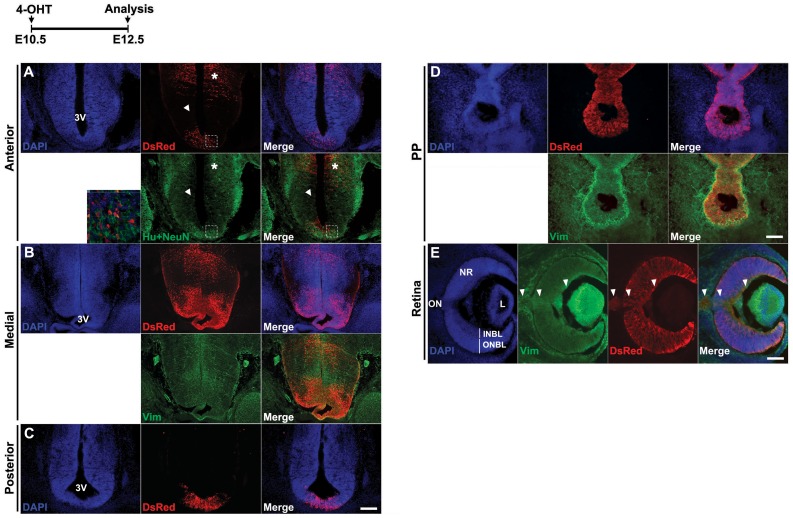
Tdtomato reporter expression is seen in neural progenitors of the ventral forebrain and retina in *Rax-CreER^T2^;Ai9* mice following 4-OHT induction at E10.5. E10.5 pregnant mice received 1.5-OHT through oral gavage and were sacrificed at E12.5. (A–C) Coronal views of the anterior, medial and posterior hypothalamus, respectively. Tdtomato positive cells, visualized by anti-DsRed immunolabeling, co-express the neuronal markers NeuN and Hu (A, dashed square), as well as the neural progenitor marker vimentin (Vim) (B). A region of ventral hypothalamic neuroepithelium by the dashed square is enlarged to show the colocalization with neuronal markers in more detail (A, bottom left panel). A white asterisk indicates the dorsal hypothalamic domain of Tdtomato expression. (D and E) Coronal view of posterior pituitary and retina. Arrowheads indicate coexpression of Tdtomato with vimentin in optic nerve (ON), which contains axons of retinal ganglion cells (RGCs), an early-born cell type. 3V, third ventricle; PP, posterior pituitary; NR, neural retina; L, lens. Sections were counterstained with DAPI (blue). Scale bars: 100 µm.

**Figure 4:**
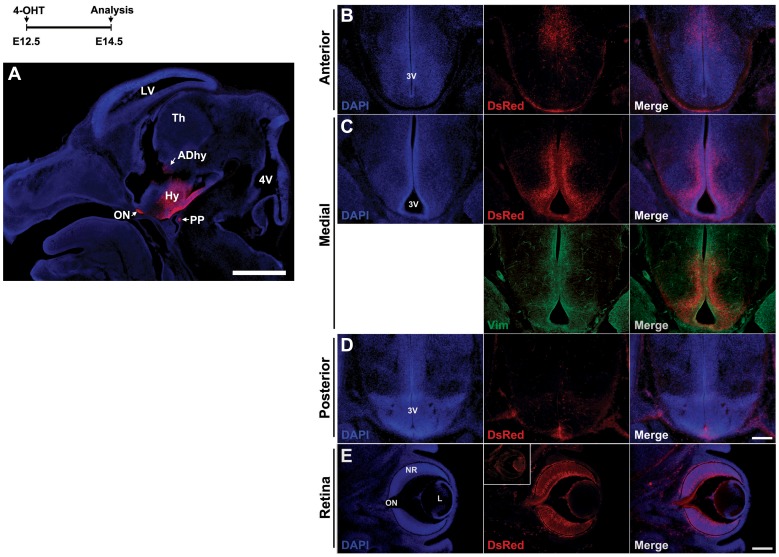
Tdtomato reporter expression is restricted to hypothalamus and retina in *Rax-CreER^T2^;Ai9* mice following 4-OHT induction at E12.5. E12.5 pregnant mice received 1.5-OHT orally through gavage and were sacrificed at E14.5. (A) Sagittal view represents the specific induction of reporter gene within the ventral hypothalamic region. LV, lateral ventricle; Th, thalamus; Hy, hypothalamus; PP, posterior pituitary; 4V, fourth ventricle; ON, optic nerve; DAhy, dorsal anterior hypothalamus. (B–D) Coronal views of the anterior, medial and posterior hypothalamus, respectively. Tdtomato expression was higher in the medial hypothalamus than either anterior or posterior and overlapped with vimentin signal along the third ventricle (3V) (C). (E) Coronal section of the retina. The weak red fluorescence observed in the hyloid vessel and in periocular tissues represents background staining, with inset showing a wildtype control eye for comparison. Red fluorescence was detected in the RGCs and their axonal projection toward the optic nerve (ON). NR, neural retina; L, lens. Sections were counterstained with DAPI (blue). Scale bars: 500 µm (A), 200 µm (B–E).

We next tested *Rax-CreER^T2^;Ai9* mice at later stages of development to determine whether 4-OHT-induced Cre activity faithfully replicated endogenous *Rax* expression patterns [12]. A single oral gavage treatment with 4-OHT at E10.5 resulted in selective induction of Tdtomato in ventral hypothalamic progenitors by E12.5 (Figure 3A), along with more limited induction in anteriodorsal hypothalamic progenitors (Figure 3A, white asterisk) and newly generated hypothalamic neurons (Figure 3A, dashed square). Hypothalamic progenitors that are located between the tuberoventral and anteriodorsal domains are unlabeled (Figure 3A, white arrowhead), and likely correspond to Dlx1/2-expressing progenitors that give rise to structures such as the dorsomedial hypothalamic nucleus, which does not express *Rax* mRNA [12], [13]. Robust labeling of the posterior pituitary (PP) was also observed (Figure 3D), along with progenitors throughout the retinal outer neuroblastic layer (ONBL) and postmitotic cells of the inner neuroblastic layer (INBL) (Figure 3E). Red fluorescence in axons in the optic nerve indicated that these were retinal ganglion cells (RGCs), one of the earliest-born retinal cell types [29]. Cre induction at E12.5, the peak period of hypothalamic neurogenesis, resulted two days later in widespread and selective labeling of ventral hypothalamic progenitor and precursor cells (Figure 4A–D). Selective labeling of neuroretina is also observed, with red fluorescence in RGC cell bodies and axons being particularly prominent (Figure 4E). This likewise closely matches the reported pattern of *Rax* expression at E12.5 [12].

**Figure 5:**
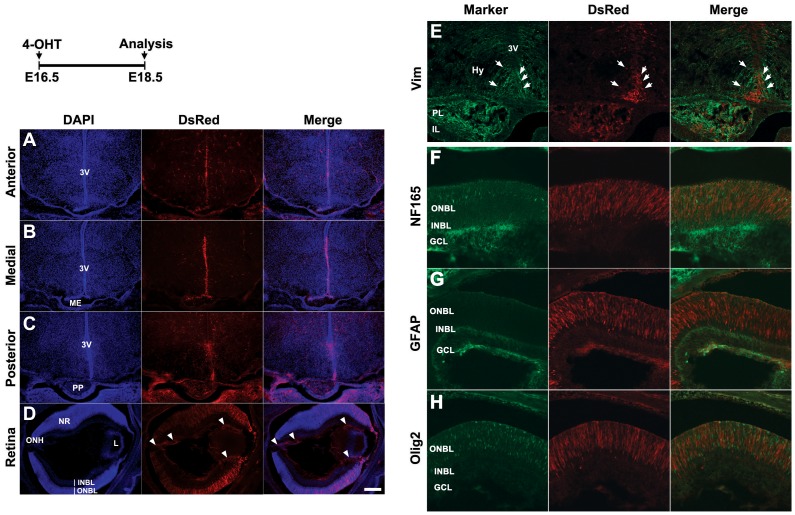
Tdtomato reporter expression is restricted to radial glia-like cells in hypothalamus, posterior pituitary, and specific retinal cell subtypes following 4-OHT induction at E16.5. E14.5 pregnant mice received 1.5-OHT orally through gavage and were sacrificed at E18.5. (A–D) Coronal views of the anterior, medial, posterior hypothalamus and retina, respectively. Reporter expression was highly limited to the radial glial cells labeled with vimentin along the third ventricular wall (A–C and E, white arrows). 3V, third ventricle; ME, median eminence; PP, posterior pituitary; NR, neural retina; ONH, optic nerve head; L, lens. (E–G) High power confocal images of hypothalamus (E) and central retina (F,G). A small number of parenchymal cells were colocalized with vimentin (E, white arrows), GFAP (F, white arrows) and Olig2 (G, white arrows) in hypothalamus (Hy). A small set of Tdtomato-positive cells in retina were colocalized with GFAP (I) and Olig2 (J) in GCL and ONBL respectively, but not with NF165 (H). IL, intermediate lobe; ONBL, outer neuroblastic layer; INBL, inner neuroblastic layer; GCL, ganglion cell layer. Sections were counterstained with DAPI (blue). Scale bars: 200 µm.

In contrast, 4-OHT injection at E16.5, after embryonic hypothalamic neurogenesis is completed, resulted in Tdtomato expression that was mostly restricted to vimentin-positive radial glia-like cells lining the ventral walls of the third ventricle (Figure 5A–C,E). A limited number of Tdtomato-positive cells were also found in the hypothalamic parenchyma (Figure 5A–C). Tdtomato expression is also observed in vimentin-positive cells of the posterior pituitary (Figure 5E). In the central retina, Tdtomato expression is primarily observed in the ONBL, with only an occasional positive cell in the INBL (Figure 5D), and colocalization of Tdtomato with the RGC marker NF165 is not observed (Figure 5H). This corresponds well with the known time course of RGC generation, which is essentially complete in the central retina by E16.5. In contrast, in the peripheral retina, more Tdtomato-positive cells and processes are seen in the INBL and some colocalization of Tdtomato is seen in the optic nerve, indicative of expression in later born RGCs of the peripheral retina (Figure 5D). In addition, colocalization of Tdtomato with GFAP-positive retinal astrocytes was seen in the INBL (Figure 5F). These do not derive from retinal neuroepithelium, but instead migrate into the retina along the optic nerve [30]. This observation suggests that that a subset of retinal astrocytes may be derived from Rax-expressing hypothalamic progenitor cells. Finally, a subset of Tdtomato-positive cells in the ONBL were double-labeled with Olig2, which is expressed in a subset of lineage-restricted retinal progenitor cells (Figure 5G) [31].

**Figure 6:**
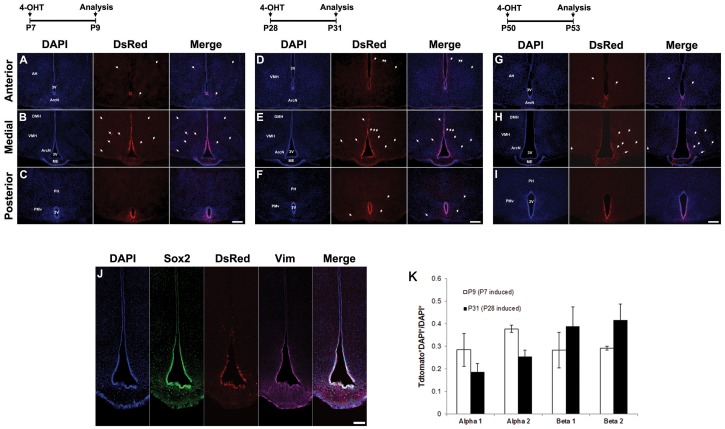
Tdtomato reporter expression in hypothalamus following postnatal 4-OHT induction in *Rax-CreER^T2^;Ai9* mice. *Rax-CreER^T2^;Ai9* mice received a single dose of 4-OHT (1.0 mg,1.5 mg and 2 mg, respectively) by intraperiotoneal injection (i.p) at three different time points, P7 (A–C), P28 (D–F) and P50(G–I), and were analyzed 2(A–C) or 3 (D–I) days following injection. (J) Representative confocal tile scan images of triple immunolabeling shows co-expression of DsRed, Sox2, and vimentin in the hypothalamic tanycytes along the third ventricular wall. AH, anterior hypothalamus; 3V, third ventricle; ArcN, arcuate nucleus; DMH, dorsomedial hypothalamus; VMH, ventromedial hypothalamus; ME, median eminence; PH, posterior hypothalamus; PM**v**, ventral premammillary nucleus. Sections were counterstained with DAPI (blue). Scale bars: 200 µm. (K) Efficiency of reporter induction in hypothalamic tanycytes in the indicated third ventricular region following a single 4-OHT injection at P7 and at P28, quantifying the data shown in 6A–C and 6D–F. A total of 2226 DsRed-positive and 7629 DAPI-positive cells were counted for this analysis, which was obtained by analyzing a total of 4 sections each from 4 *Rax-CreER^T2^;Ai9* mice for each condition tested. Standard deviation is shown. Two-way ANOVA analysis did not reveal any significant (p<0.05) differences in efficiency of reporter induction among tanycytes in any of the four different zones indicated.

**Figure 7:**
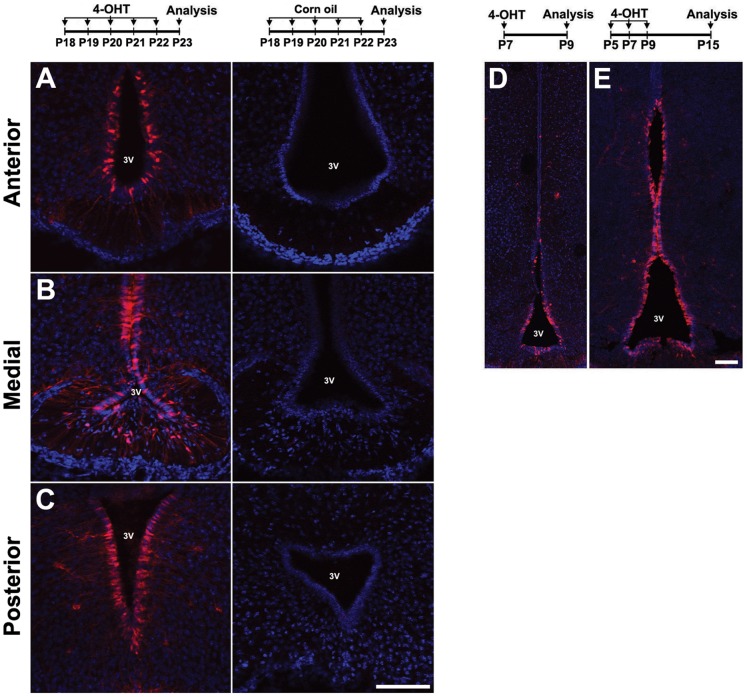
Specificity and efficiency of induction of TdTomato reporter in hypothalamus following postnatal 4-OHT induction in *Rax-CreER^T2^;Ai9* mice. (A–C) Comparison of hypothalamic reporter expression between uninduced oil-treated control (right panels) and 4-OHT induced mice (left panels). P18 *Rax-CreER^T2^;Ai9* mice received i.p. injections daily with either oil vehicle or 4-OHT (0.3 mg at P18–20 and 1.0 mg at P21–22) for 5 days and were analyzed at P23. Control mouse brains did not have morphological defects and did not show any ectopic reporter expression in the anterior (A), medial (B) or posterior hypothalamus (C). (D–E) Efficiency of induction with a single dose (1.0 mg of 4-OHT) at P4 (D) or three daily doses (0.3 mg of 4-OHT) each at P5, P7, and P11 (E). Multiple injections resulted in enhanced induction compared to single injection. Sections were counterstained with DAPI (blue). Scale bars: 200 µm (F).

We next investigated Cre activity at three postnatal time points. In the hypothalamus, Tdtomato expression is restricted to hypothalamic tanycytes following 4-OHT intraperitoneal (i.p.) injection at P7, P28 and P50 (Figure 6A–I), which again recapitulates the previously reported pattern of *Rax* mRNA expression [4], [12], [32]. Td tomato labeled cells robustly expressed Sox2 and vimentin, both of which are prominently expressed in tanycytes (Figure 6J). TdTomato expression is not observed in more dorsally located ependymal cells of the third ventricle at any postnatal stage. Tanycytes are more efficiently labeled at P7 and P28 than at P50 (Figure 6A–I). At both P7 and P28, efficient induction of Tdtomato expression is observed in ventricular zones that α1, α2, β1 and β2 tanycytes (Figure 6K). Daily i.p. injections of corn oil alone from P18–22 do not result in any Tdtomato labeling of tanycytes in *Rax-CreER^T2^;Ai9*mice (Figure 7A–C right panels). Considerably more tanycyte labeling was seen following a series of three 4-OHT injections from P5–P9 when compared to a single injection at P7 (Figure 7D,E).

**Figure 8:**
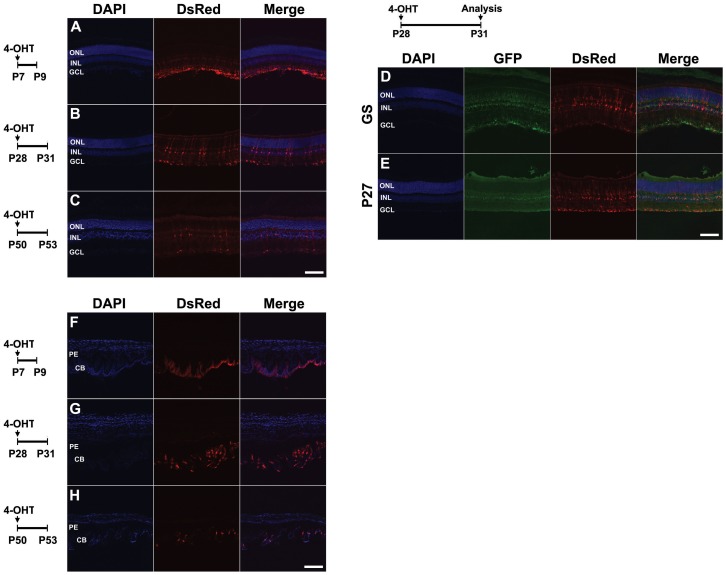
Tdtomato reporter expression in retina following postnatal 4-OHT induction in *Rax-CreER^T2^;Ai9* mice. *Rax-CreER^T2^;Ai9* mice received 4-OHT (1.0 mg,1.5 mg and 2 mg, respectively) i.p at three different time points, P7 (A,F), P28 (B,D,E,G), and P50(C,H), and their neural retinas (A–E) and ciliary margins (F–G) were analyzed after 2(A,F) or 3 (B–E, G–H) days. A single i.p. injection successfully induced reporter expression in radial Müller glia in all three different postnatal stages tested, particularly at P31. Tdtomato co-labeled with the Müller glia markers GS (D) and P27 (E). Interestingly, reporter expression was also observed in the non-pigmented zone of the ciliary margin at all indicated postnatal stages (F–H). ONL, outer nuclear layer; INL, inner nuclear layer; GCL, ganglion cell layer; PE, pigmented epithelium; CB, ciliary body. Sections were counterstained with DAPI (blue). Scale bars: 200 µm.

In the neuroretina, robust and selective Cre activation is seen in radial Müller glia following 4-OHT i.p. injection at P7 (Figure 8A), likewise reflecting the previously reported developmental pattern of *Rax* mRNA expression [18], [28], [33]. Surprisingly, despite the fact that only very low levels of *Rax* mRNA expression have been reported in mature Müller glia [28], [34], [35], we also observe a less efficient but highly selective induction of Tdtomato in Müller glia of animals injected at both P28 and P50 (Figure 8B–E). Finally, at all three stages, we also observe robust and selective induction of the reporter in the non-pigmented zone of the ciliary body (Figure 8F–H).

**Figure 9:**
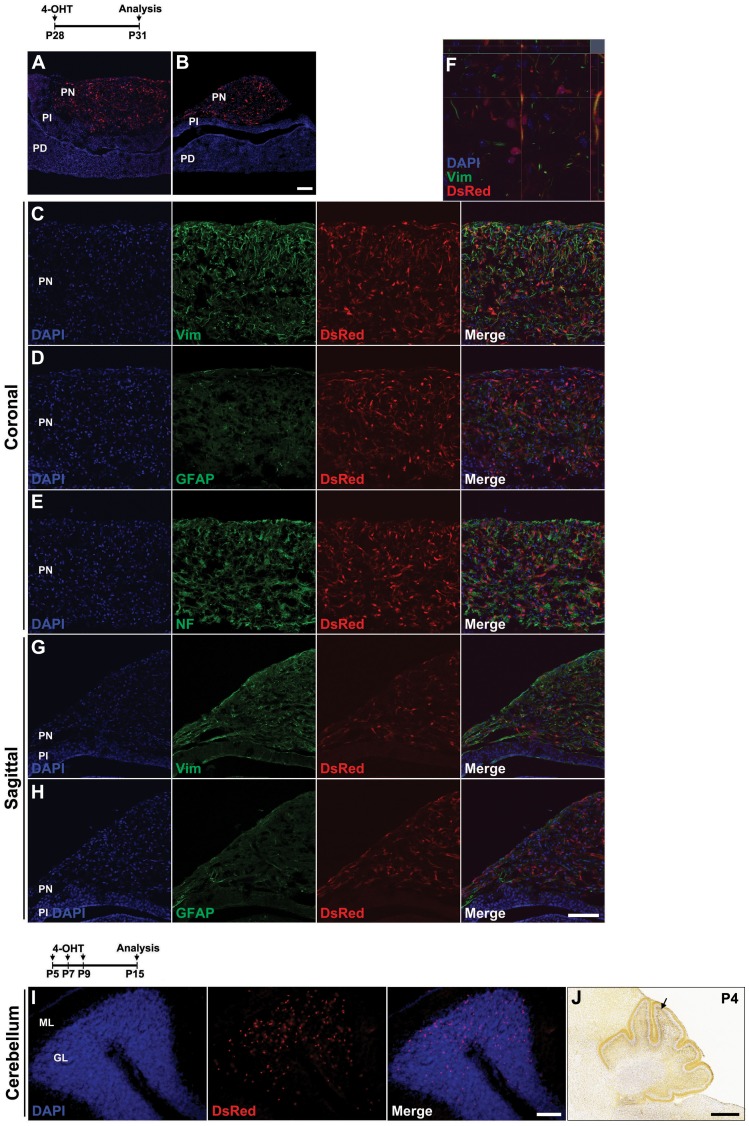
Tdtomato reporter expression in posterior pituitary and cerebellum following postnatal 4-OHT induction in *Rax-CreER^T2^;Ai9* mice. (A–H) *Rax-CreER^T2^;Ai9* double heterozygote mice received 1.5 mg of 4-OHT at P28 and their pituitary glands were analyzed 3 days later. Coronal (A) and sagittal (B) sections stained with DsRed. Reporter expression was restricted in only pars nervosa (PN). PI, pars intermediate; PD, pars distalis. (C–F and G–H) High power confocal images of (A) and (B), respectively. A subset of Tdtomato-positive cells colocalized with vimentin (C, F), but not with GFAP or NF165. (I) *Rax-CreER^T2^;Ai9* double heterozygote mice received 0.3 mg of 4-OHT three times at P5, P7, and P9, and their cerebellums were analyzed at P15. Reporter-positive cells were prominently robust in the granular layer (GL). (J) *Rax* mRNA *in situ* hybridization signal in cerebellum at P4.5 (data obtained from the Allen Brain Atlas), with the flocculus indicated by a black arrowhead. Sections were counterstained with DAPI (blue). ML, molecular layer. Scale bars: 200 µm (A–H), 400 µm (J).

In addition, we observed robust and selective labeling of a subset of cells in the posterior pituitary following 4-OHT i.p. injection at P28 (Figure 9A–B). These cells are immunopositive for vimentin, but not GFAP or neurofilament, indicating that they are most likely fibrous pituicytes [36], or other glial cells of the neurohypophysis (Figure 9C–H) [36], [37]. Surprisingly, we also observed Tdtomato labeling in a subset of cells in the granule layer of the flocculus of the cerebellum when 4-OHT was delivered between P5 and P9 (Figure 9I), though not at any point earlier or later. Though not previously reported in the literature, inspection of *in situ* hybridization data obtained from the Allen Brain Atlas revealed that *Rax* mRNA was indeed selectively expressed in the flocculus at P4 but not at later time points (Figure 9J). Finally, we examined other organs outside the brain of the *Rax-CreER^T2^;Ai9* mice, including skeletal muscle, heart, kidney, liver, intestine, and adrenal, but did not find reporter expression in any other tissue (data not shown).

## Discussion

This study has identified *Rax-CreER^T2^* knock-in mice as potentially useful for studies of both retinal and hypothalamic development, as well as for studying the biology of Müller glia and tanycytes. Heterozygous *Rax-CreER^T2^* knock-in mice are phenotypically normal and show no ectopic Cre activation. In addition, the pattern of 4-OHT induced Cre activity precisely recapitulates the expression pattern of the endogenous *Rax* gene. Indeed, we find that this line is selectively active in mature Müller glia and in cells of the ciliary margin, which express *Rax* mRNA at very low levels [38]–[40]. We anticipate that this mouse line will be particularly useful for analyzing temporally dynamic functions of genes that are important for retinal development; examples include homeodomain factors such as *Pax6*, *Chx10* and *Lhx2*. These factors are both broadly expressed in RPCs and also thought to act in discrete temporal windows to control specification of individual retinal cell types [41]–[45]. It will also likely prove useful for studies of the genetic mechanisms guiding hypothalamic cell specification during embryonic development. Because Cre is selectively active in Müller glia and tanycytes, both of which may act as neural progenitors in adults [18], [46]–[48], this mouse line makes it possible to prospectively label these cells for lineage analysis and determine the fates of the cells they generate. Furthermore, this mouse line can be used to selectively disrupt the function of genes that are candidates for regulating proliferative and neurogenic potential in both radial glial cell types. The selective expression of Tdtomato in the developing posterior pituitary and mature pituicytes of this mouse line will aid research aimed at understanding the development and function of these poorly studied cell populations.

## Materials and Methods

### Ethics statement

All mice used in these studies were maintained and euthanized according to protocols approved by the Institutional Animal Care and Use Committee at the Johns Hopkins School of Medicine.

### Mice

C57BL/6J wild type mice were purchased from Charles River and were bred with chimeric mice to determine germline transmission of the *Rax-CreER^T2^* targeted locus. The neomycin cassette was removed using FLPeR mice [26] donated by Dr. Jeremy Nathans (Johns Hopkins). The FLPeR was bred out of the mouse line after the neomycin cassette elimination. *R26-CAG-lox-stop-lox-TdTom* (*Ai9*) mice were a generous gift from Dr. Xinzhong Dong (Johns Hopkins).

### Construction of *Rax-CreER^T2^* targeting and control vectors

We used recombineering to retrieve a 14 kb fragment from a C57BL/6 background Bacterial Artificial Chromosome (BAC) containing the *Rax* locus. Briefly, SW106 cells containing the BAC were electroporated with a pGK-DTa vector containing two previously cloned 500 bp retrieving arms (a 3′ arm and a 5′ arm) homologous to the ends of the chosen 14 kb BAC region. In parallel, we generated a control vector with a longer short arm, so as to allow testing of PCR primers used in screening ES cells with correctly integrated targeting cassettes. The control vector was designed with a 500 bp 3′ arm that was homologous to the BAC region 500 bp downstream from the 3′ arm used in the construction of the CreER^T2^ targeting vector.

We activated the recombineering machinery of the SW106 cells (containing the BAC and the pGK-DTa vector) as described [49], which induced the retrieval of the 14 kb BAC region by homologous recombination between the BAC and the 3′ and 5′ 500 bp arms of the pGK-DTa vector. The resulting vector (pGKDTa-LA-Rax-SA) consisting of the *Rax* locus (4.5 kb), an upstream 7.5 kb long arm (LA), a short 2 kb arm (SA) and the negative selection cassette pGK-DTa, was then used for a second round of recombineering with the vectors containing the CreER^T2^-neo targeting constructs. The *Rax-CreER^T2^*-neo constructs were generated using traditional cloning as follows: first, we cloned two 500 bp arms homologous to genomic sequence upstream (5′ arm) and downstream (3′ arm) from the eighth nucleotide downstream of the first base in the initiator methionine codon of *Rax* (UCSC genome browser July 2007 (NCBI37/mm9) Assembly) into pBluescript. We next introduced a neomycin cassette between these two arms. We then cloned the CreER^T2^ (obtained from Addgene – pCAG-CreER^T2^, Plasmid 14797) upstream of the neomycin cassette, to generate the CreER^T2^-neo construct. The resulting CreER^T2^-neo cassette was inserted into the *Rax* locus by homologous recombination between the 500 bp arms of the CreER^T2^-neo construct and the pGK-DTa-LA-Rax-SA vector.

The control construct was generated in the same way as the targeting vectors, except that the control construct contained only the neomycin cassette and its short arm was 1 kb longer. A longer short arm allowed the standardization of a PCR protocol to test whether the 3′ portion of the targeting construct had been correctly inserted into the endogenous *Rax* locus. In this PCR protocol, the forward primer targeted the neomycin cassette and the reverse primer targeted the second intron of *Rax*; a 3 kb fragment was produced only when the constructs were inserted in the correct location. PCR primer pairs suitable for identifying the correctly inserted short arm were then identified using the control construct as a template. Following this, the Rax-CreER^T2^-neo targeting vector was fully sequenced, confirmed as correctly designed. This vector was then submitted to the Johns Hopkins ES Cell Core Facility for electroporation into MC1 mouse ES cells derived from 129S6/SvEvTac, using previously described protocols [50].

### Screening of electroporated ES cells

Embryonic stem cell colonies electroporated with either the targeting construct Rax-CreER^T2^ or the control construct. These were then screened for correct locus insertion by PCR amplification of a 3 kb DNA fragment using a forward primer located in the neomycin cassette 5′-CTTCTATCGCCTTCTTGACG-3′ (P1) and a reverse primer located in chromosome 18 within the second intron of the *Rax* gene forward 5′-TTTGTCATTTCCCCTCGTAG-3′ (P2). This genotyping was performed using the following PCR touchdown protocol: 95°C 5 min, 94°C 30 min, 70°C 1 min; minus 1°C each cycle (15 times), 72°C 3 min, 94°C 30 sec, 55°C 30 sec for 30 cycles, 72°C 3 min, 72°C 7 min, 4°C hold.

### Southern blotting

ES cell colonies that tested positive by PCR for correct 3′ insertion into the Rax locus and the ES cell colonies electroporated with the control vector were grown further. DNA was extracted using the Blood & Cell Culture DNA Mini Kit from Qiagen (Cat. 13323), following the manufacturer's protocol. Purified DNA was digested with AfIII and SexAI (*Rax-CreER^T2^*-neo construct) and used for Southern blotting. We designed a 511 bp probe amplified by PCR from a wild type C57BL/6J DNA using the following primers: forward 5′-ATGCATTTAGATGCCTGATTGCCAAT-3′ and reverse 5′-ACGCGTCAAAACCACAGTAAACCAAG-3′. This probe hybridized 5.4 kb upstream of the long arm of the targeted locus. The probe labeling and Southern blot were performed using the DIG-High Prime DNA Labeling and Detection Starter Kit I from Roche (Cat. 11745832910) following the manufacturer's protocol. Karyotyping and ES cells pronuclear injection into ICR host embryos were performed at the Johns Hopkins Transgenic core as previously described [50].

### Genotyping

DNA for mouse genotyping was obtained from tail tips collected carefully with fresh razor blades to avoid cross contamination. Tails were incubated at 55°C overnight in lysis solution containing proteinase K (0.1 µg/ul). DNA extraction was performed as previously described [25].

After establishing the *Rax-CreER^T2^* mouse line, the presence of the *Rax-CreER^T2^* allele was determined through genotyping for Cre recombinase using the following primers: forward 5′-TTCCCGCAGAACCTGAAGAT- 3′ (P3) and reverse 5′-CCCCAGAAATGCCAGATTAC-3′ (P4) which produced a 350 bp PCR product. The Cre PCR genotyping protocol was: 94°C for 5 min, 94°C for 30 sec, 55°C for 30 sec, 72°C for 1.3 min for 30 cycles, 72°C for 5 min, 4°C hold.

Removal of the neomycin cassette from the *Rax-CreER^T2^; ROSA26-FLPeR* mice was confirmed by PCR using the following primers: forward 5′-GGCGCGAGCCCTGATGCTC-3′ (P5) and reverse 5′-TTGGGTGGAGAGGCTATTCGGCTATGAC-3′ (P6), which produced a 459 bp PCR product. The PCR protocol used was: 94°C for 5′, 94°C for 30 sec, 63°C for 30 sec, 68°C for 45 sec for 30 cycles, 72°C for 5 min, 4°C hold.

Genotyping for the Ai9 transgene was done using the forward primer 5′-CTGTTCCTG TACGGCATGG-3′, and the reverse primer 5′-GGCATTAAAGCAGCGTATCC-3′ which produced a 296 bp PCR product. The PCR protocol was: 94°C 5 min, 94°C 30 sec, 55°C 45 sec, 72°C 45 sec for 35 cycles, 72°C 7 min and 4°C hold.

### Cre induction by 4-hydroxytamoxifen in *Rax-CreER^T2^;Ai9* mice

4-OHT was prepared by sonicating 50 mg of 4-OHT (Sigma) in 1.5 mL of 100% ethanol at 33°C. 90 µl of 4-OHT/ethanol was vortexed with 300 µl of corn oil, and ethanol removed by vacuum centrifugation. Transgenic mice, double heterozygous for Rax-CreER^T2^ and Ai9, were given varying doses of 4-OHT depending on the mouse age (see Table 1). Cre activation in embryonic mice was induced through oral gavage of the mother. Postnatal mice were given 4-OHT by i.p. injections.

### Tissue collections and histology

Mice were harvested at multiple embryonic and postnatal time points. For embryonic mice, gestational age was determined by counting the morning of the vaginal plug detection as embryonic day 0.5 (E0.5). Crown to rump lengths were also checked to verify gestational age. Embryos were dissected and fixed in 4% paraformaldehyde (PFA) in 1×PBS (w/v) overnight at 4°C. Embryos were then washed three times with PBS and immersed in 30% sucrose in 1×PBS (weight/volume) at 4°C for several days. Postnatal mice were anesthetized using a cocktail of ketamine, acepromazine, and xylazine (9∶9∶3 volume ratio), and transcardially perfused with 4% PFA. The brain, eyes, and pituitary gland were removed and post-fixed overnight at 4°C with 4% PFA on a rotating platform, followed by 24 hours immersion in in 30% sucrose/1× PBS. Tissues were then embedded in OCT (Tissue Tek, Sakura Finetek), following which either 25 µm (for embryonic tissue) or 40 µm (for postnatal tissue) sections were cut on a cryostat.

### Immunohistochemistry

All embryonic tissues were placed directly on Superfrost Plus slides (Fisher). Postnatal tissues were also directly placed on the slide for IHC except when staining for mouse anti-HuC/D. For HuC/D detection, free floating sections were first cut into individual wells in a 24 well plate containing 1× PBS (Falcon), then mounted onto Superfrost Plus slides. Tissue sections were fluorescently immunostained for the following markers: Tdtomato: 1∶500 rabbit polyclonal anti-DsRed (Living Colors), 1∶500 rat monoclonal anti-DsRed (Antibodies Online), neuronal markers: 1∶200 mouse monoclonal anti-HuC/D (Invitrogen), 1∶500 mouse monoclonal anti-NF165 (Developmental Studies Hybrididoma Bank), 1∶500 mouse anti-NeuN (Chemicon), tanycyte and/or glial progenitor cell markers: 1∶1000 rabbit polyclonal anti-GFAP (DAKO), 1∶1500 chicken polyclonal anti-vimentin (Millipore), 1∶500 rabbit polyclonal anti-Sox2 (Millipore), Müller glia markers: mouse monoclonal anti-P27 (Invitrogen), mouse monoclonal anti-GS (BD Bioscience), oligodendrocyte marker: 1∶500 rabbit polyclonal anti-Olig2 (Millipore). The AlexaFluor tagged secondary antibodies used were Alexa488 (green), Alexa555 (red), and Alexa633 (far red) (Molecular Probes). DAPI was used as a nuclear counterstain (Molecular Probes).

### Image acquisition

Single plane and *z*-stack confocal images were obtained in a Zeiss LSM510 Meta confocal microscope equipped with Zen 2009 software and using a 10×, 20× or 63× objective and digital zoom 1. Images for each time point were acquired under identical conditions, using the same pinhole diameter, gain and contrast. Images for the different treatments were analyzed qualitatively for the presence or absence of signal and for co-labeling with cell-specific markers.
