# Correction: *Rax-CreER^T2^* Knock-In Mice: A Tool for Selective and Conditional Gene Deletion in Progenitor Cells and Radial Glia of the Retina and Hypothalamus

**DOI:** 10.1371/journal.pone.0102875

**Published:** 2014-07-10

**Authors:** The PLOS ONE Staff

The third author's name is spelled incorrectly. The correct name is: Ana L. Miranda-Angulo. The correct citation is: Pak T, Yoo S, Miranda-Angulo AL, Wang H, Blackshaw S (2014) *Rax-CreER^T2^* Knock-In Mice: A Tool for Selective and Conditional Gene Deletion in Progenitor Cells and Radial Glia of the Retina and Hypothalamus. PLoS ONE 9(4): e90381. doi:10.1371/journal.pone.0090381

## References

[1] Pak T, Yoo S, Miranda-Angulo AM, Wang H, Blackshaw S (2014) *Rax-CreER^T2^* Knock-In Mice: A Tool for Selective and Conditional Gene Deletion in Progenitor Cells and Radial Glia of the Retina and Hypothalamus. PLoS ONE 9(4): e90381 doi:10.1371/journal.pone.0090381 24699247 10.1371/journal.pone.0090381 PMC3974648

